# Impact of Late and Recurrent Acute Graft Pyelonephritis on Long-Term Kidney Graft Outcomes

**DOI:** 10.3389/fimmu.2022.824425

**Published:** 2022-03-02

**Authors:** Margaux Pacaud, Luc Colas, Clarisse Kerleau, Florent Le Borgne, Magali Giral, Sophie Brouard, Jacques Dantal

**Affiliations:** ^1^ INSERM, CHU Nantes, Nantes Université, Centre de Recherche en Transplantation et Immunologie UMR1064, ITUN, Nantes, France; ^2^ CHU Nantes, Nantes Université, Service de néphrologie – immunologie clinique, Nantes, France; ^3^ Université de Tours, INSERM UMR 1246-SPHERE, Nantes, France; ^4^ IDBC-A2COM, Pacé, France; ^5^ Labex IGO, Nantes, France; ^6^ Centre d’Investigation Clinique en Biothérapie, Centre de ressources biologiques (CRB), Nantes, France

**Keywords:** acute pyelonephritis, kidney allograft, graft failure, patient survival, long-term outcome

## Abstract

**Background:**

While Urinary tract infections are the most common infections in kidney transplant recipients, the impact of late acute graft pyelonephritis (AGPN) on graft outcomes remains unknown. Our study was performed to more precisely evaluate the long-term impact of AGPN.

**Methods:**

We included 9052 kidney and combined kidney-pancreas recipients who underwent transplantation between 2008 and 2018 from a French multicenter cohort. The relationships between AGPN and patient and graft survival were analyzed with a time-dependent multivariate Cox model.

**Results:**

The cumulative incidence of AGPN was 20.9%. A first episode of early AGPN is associated with a non-significant increase in the risk of graft failure (hazard ratio [HR], 1.27; 95% confidence interval [95% CI], 0.90 to 1.79). Though, cumulative number of AGPN episodes (HR = 1.51; 95% CI, 0.89 to 2.57 for two episodes and HR = 2.08; 95% CI, 1.17 to 3.69 for three or more episodes) is associated with an increased risk of graft failure. In contrast, when the first episode of AGPN occurred late (i.e., 6 months post transplantation), the risk of graft failure is significantly increased (HR = 2.25; 95% CI, 1.65 to 3.07), and this risk remains relatively stable with the recurrence of late AGPN episodes. The onset of late AGPN were also associated with a higher risk of patient death.

**Conclusion:**

This analysis shows that late AGPN and recurrent AGPN are both risk factors for a poor long-term graft outcome and mortality. Late AGPN should not be considered benign infections in post-transplantation follow-up.

## Highlights

Acute graft pyelonephritis (AGPN) is common infection in kidney transplant recipient. While the impact of early AGPN on graft outcomes is well documented, the impact of late AGPN remains unknown. In a French multicentric kidney transplant cohort (DIVAT), the authors assessed the impact of late and recurrent AGPN occurring six months after transplantation on patient survival, graft survival and combined patient-graft survival, in a time manner. They report that late AGPN and recurrent late AGPN are both risk factors for a poor long-term graft outcome and mortality. These infections should therefore not be considered benign and as soon as the first episode of AGPN occurs, patients should be closely monitored to limit the risk of recurrence.

## Introduction

Kidney transplantation remains the treatment of choice for end-stage renal disease. However, this requires long-term immunosuppressive treatment that can lead to infectious complications (1). Because of the surgical procedures involved, urinary tract infections (UTIs) are the most common infections in kidney transplant recipients. UTIs include mainly presenting as cystitis and acute graft pyelonephritis (AGPN). AGPN occurs in 10 to 20% of kidney transplant patients during follow-up (2, 3). This complication requires hospitalization for close monitoring with a risk of progression to bacteremia because UTIs are the most common source of bloodstream infection (4). Moreover, interstitial infiltration of lymphocytes into the graft may constitute a warning sign, activating an alloimmune response and can thus participate to acute or chronic rejection (5). Several risk factors for AGPN have been identified, including female sex, ureteral stent, cytomegalovirus (CMV) infection, recurrent UTIs and mycophenolate mofetil treatment (6, 7). However, the impact of one or more episodes of AGPN on the renal transplant outcome is still controversial. Some reports suggested that long-term patient and graft survival were not affected by AGPN (2, 8, 9). In contrast, others have shown that early-onset AGPN is associated with the deterioration of renal function over ten years, independent of whether acute rejection occurs (7). Similarly, at least one AGPN episode occurring in the first three months after transplantation was found to be associated with a poor graft outcome, but interestingly, this deleterious effect on graft survival was no longer found if all episodes of AGPN (i.e., early and late episodes) were taken into account (6). Late-onset UTI occurring six months post transplantation seemed to correlate with an increased risk of graft loss and patient death, but until now, studies have reached inconsistent conclusions (10).

The impact of UTIs, particularly late episodes of AGPN, on kidney transplant recipients remains to be clarified. Our study therefore was performed to assess the impact of AGPN, with a special focus on late episodes of AGPN occurring six months after transplantation, on long-term kidney graft outcomes and mortality in a French multicentric kidney transplant cohort (DIVAT).

## Patients and Methods

### Population

Data were extracted from the French multicentric, observational and prospective DIVAT cohort (www.divat.fr, CNIL final agreement, decision DR-2015-087 N°914184). A total of 9052 adult kidney transplant recipients (who received a first or second single kidney transplant or a simultaneous kidney-pancreas transplant) were recruited and included from January 2008 to December 2018. Follow-up was performed in in Nantes, Paris (Necker and Saint-Louis). This study was performed in accordance with the Declaration of Helsinki. All participants enrolled in this study signed informed consent forms.

### Definition of AGPN

AGPN was defined by the simultaneous presence of fever (after exclusion of others causes of fever) with a positive urine culture (bacteriuria count>10 (5) colony-forming units/ml), and/or bacteremia, accompanied by one or more of clinical symptoms or biological abnormalities; painful graft, chills, cystitis, dysuria, increased serum creatinine and hyperleukocytosis [adapted from (2, 6)]. To homogenize our data, the episodes of pyelonephritis are grouped under the same code.

### Immunosuppressive Treatment and Prophylaxis

According to the guidelines, patients received induction treatment at the time of transplantation with ATG (thymoglobulin (n=5752), 1.5 mg/kg/day (maximum 75 to 100 mg/day) or basiliximab (n=3081) (20 mg Simulect on day 0 and day 4), depending mainly on immunization status and/or transplantation rank. All patients received initial corticosteroid therapy, with at least one preoperative methylprednisolone bolus, followed by maintenance immunosuppressive treatment. Details of the immunosuppressive regimens are given in **Table 1**. All patients received prophylaxis against *Pneumocystis jirovecii* pneumonia during the first six months of transplantation, and valganciclovir was administered for at least 3 months except in the case of negative serology results for both donors and recipients.

**Table 1:** Characteristics of 9052 transplant recipients included in the analysis.

	Whole sample (n=9052)			No AGPN (n=7286)				First AGPN in the first 6-months post-transplantation (n=954)			First AGPN after 6-months post-transplantation (n=812)		
	NA	n	%	NA	n	%		NA	n	%	NA	n	%
**Recipient characteristics**													
Recipient age (SD)	0	51.0 (14.1)		0	50.6 (14.1)			0	53.5 (14.3)		0	51.5 (14.3)	
Recipient age ≥55 years	0	4001	44.2	0	3110	42.7		0	517	54.2	0	374	46.1
Retransplantation	0	1309	14.5	0	1007	13.8		0	154	16.1	0	148	18.2
Male sex	0	5743	63.4	0	4963	68.1		0	452	47.4	0	328	40.4
Kidney transplantation (vs SPK transplantation)	0	8508	94.0	0	6840	93.9		0	898	94.1	0	770	94.8
Renal replacement therapy	30			29				0			1		
• Hemodialysis		6749	74.8		5408	74.5			731	76.6		610	75.2
• Peritoneal dialysis		824	9.1		682	9.4			76	8.0		66	8.1
• Preemptive transplantation		1449	16.1		1167	16.1			147	15.4		135	16.6
Recurrent initial nephropathy	0	2209	24.4	0	1855	25.5		0	191	20.0	0	163	20.1
History of diabetes	0	2179	24.1	0	1729	23.7		0	238	24.9	0	212	26.1
Urological history	0	999	11.0	0	722	9.9		0	149	15.6	0	128	15.8
Use of ureteral stent	245	7246	80.0	223	5844	80.2		14	775	81.2	8	627	77.2
Positive recipient CMV serology	109	5867	65.6	87	4724	65.6		7	636	67.2	15	507	63.6
**Donor characteristics**													
Deceased donor	0	7510	83.0	0	5963	81.8		0	834	87.4	0	713	87.8
Donor age (SD)	32	52.1 (16.4)		28	51.6 (16.3)			4	54.9 (16.2)		0	53.2 (16.6)	
Donor age ≥55 years	32	4209	46.7	28	3280	45.2		4	516	54.3	0	413	50.9
Male donor	12	5044	55.8	12	4049	55.7		0	539	56.5	0	456	56.2
Positive donor CMV serology	22	4964	55.0	19	3968	54.6		1	544	57.1	2	452	55.8
Cold ischemia time ≥18 hours	83	2653	29.6	70	2041	28.3		7	321	33.9	6	291	36.1
**Immunosuppression and prophylaxis at transplantation**													
Cold ischemia (SD)	83	14.5 (8.1)		70	14.2 (8.1)			7	115.7 (8.0)		6	15.9 (8.0)	
Depleting induction at transplantation	43	5752	63.8	40	4602	63.5		3	623	65.5	0	527	64.9
Maintenance immunosuppressive treatment at transplantation													
• Ciclosporine	43	1594	17.6	40	1290	17.7		3	156	16.6	0	148	18.22
• Tacrolimus	43	7407	82.2	40	5947	82.1		3	796	83.7	0	664	81.8
• mTOR inhibitors	43	342	3.7	40	285	3.9		3	39	4.1	0	24	3.0
• Anti-proliferatives	43	8713	96.2	40	6993	96.0		3	924	96.9	0	796	98.0
• Oral corticoids	43	8635	95.39	40	6965	95.6		3	907	95.1	0	763	94.0
CMV prophylaxis treatment at transplantation	424	5722	66.3	381	4584	66.4		23	646	69.4	20	492	62.1
**Immunology**													
Immunization pre-transplantation	1019			831				90			98		
• Positive anti-HLA Ab no DSA		3410	42.4		2719	42.1			369	42.7		322	45.1
• Positive DSA		1061	13.2		829	12.8			149	17.2		83	11.6
• Negative anti-HLA Ab		3562	44.3		2907	45.0			346	40.0		309	43.3
HLA-A-B-DR mismatches > 4	80	1660	18.5	69	1336	18.5		8	193	20.4	3	131	16.2
Pregnancy history	1039	1734	21.6	709	1217	18.5		149	273	33.9	181	244	38.7
**Post-transplantation outcomes**													
Delayed graft function	589	1878	22.2	549	1422	21.1		20	272	29.1	20	184	23.2
At least one rejection	0	2018	22.3	0	1524	20.9		0	253	26.5	0	239	29.4
Number of rejection (SD)	0	0.3 (0.7)		0	0.3 (06)				0.4 (0.7)			0.4 (0.9)	
Treatment of rejection	0	2018	22.3	0	1526	20.9		0	253	26.5	0	239	29.4
• No treatment	0	546	6.0	0	423	5.8		0	63	6.6	0	60	7.3
• Oral corticoids	0	890	9.8	0	658	9.0		0	116	12.8	0	116	14.2
• Anti-thymoglobulin	0	81	0.9	0	74	1.0		0	7	0.7	0	0	0
• Others treatments (IVIg, Rituximab, plasmapheresis)	0	501	5.5	0	371	5.1		0	67	7.0	0	63	7.7
At least one CMV infection	0	1314	14.5	0	962	13.2		0	186	19.5	0	166	20.4
Cause of death	0	758	8.3										
• Cardio-vascular	0	172	1.9										
• Malignancy	0	192	2.1										
• Infections	0	160	1.8										
• Others	0	96	1.1										
• Undetermined	0	138	1.5										

AGPN, acute graft pyelonephritis; CMV, cytomegalovirus; CNI, calcineurine inhibitor; DSA, donor-specific antibodies; HLA, human leucocyte antigens; SD: standard deviation; NA, not available (missing).

### Clinical and Biological Data

The following recipient characteristics were collected: age, sex, transplantation rank, initial renal disease, renal replacement therapy, history of diabetes, urological history often associated with recurrent UITs prior to kidney transplantation (nephrectomy and/or surgical procedures on urinary tract for stenosis, reflux or malformative uropathy), the use of ureteral stent during transplantation (removal at 4-6 weeks after transplantation), pregnancy history, recipient CMV serology and immunization [anti-human leukocyte antigen (HLA) and donor-specific antibodies (DSAs)]. The collected donor features were age, sex, donor type (living or deceased) and CMV serology. The baseline transplantation parameters considered were transplantation type (kidney or combined kidney and pancreas), cold ischemia time, number of HLA A-B-DR incompatibilities, induction therapy, delayed graft function (DGF), recipient transfusion, maintenance treatment and CMV prophylaxis. The collected post transplantation parameters were acute rejection (one or more) and CMV infection. The follow-up and the collection of data stopped upon a patient’s return to dialysis or death.

### Outcomes

The main outcome was death-censored graft survival, defined as the time between transplantation and the first event before a return to dialysis or preemptive retransplantation. We additionally investigated patient and graft survival by considering death as an event, and patient survival was defined as the time between transplantation and death with a functioning graft. We also reported the cumulative probability of the occurrence of the *de novo* appearance of DSA, defined as the time between transplantation and the first appearance of DSA post transplantation. Finally, we studied the evolution of the estimated glomerular filtration rate (eGFR), estimated by the Modification of Diet in Renal Disease (MDRD) formula, considering all patients as Caucasian, as ethnicity was not available in our database.

### Statistics

The median duration of follow-up was estimated with a reverse Kaplan-Meier analysis (11). The cumulative incidence curves of the four binary outcomes were obtained by the Aalen-Johansen estimator, considering death and/or graft failure as competing events (12). Multivariable cause-specific time-dependent Cox models stratified by center were used to estimate the relationship between the incidence of AGPN and the studied times-to-event. Strategy of covariate selection was based on the associations between covariates and the outcome as recommended in the literature (13–15). Of note, the rejection covariate included in the survival models was a binary time-dependent covariate equal to 0 from the transplantation until the first acute rejection episode and equal to 1 thereafter. Competing events were right-censored (16). The hazard proportionality assumption was tested based on the Schoenfeld residuals (17). If this assumption did not hold, two different periods were considered. For baseline continuous covariates, the log-linearity assumption was checked with univariate analysis if the Bayesian information criterion was not reduced using natural spline transformation compared to the inclusion of the covariate in its natural scale. In the case of the violation of the assumption, variables were categorized.

To explore the associations of the early (i.e., occurring in the first 6 months post transplantation) or late (i.e., occurring more than 6 months post transplantation) occurrence of the first episodes of AGPN and the association of the total number of AGPN episodes with the outcomes, we included AGPN in the model as a time-dependent variable with seven categories (no AGPN, one early episode of AGPN, two episodes of AGPN with at least the first occurring early, three or more episodes of AGPN with at least the first occurring early, one late episode of AGPN (none early), two late episodes of AGPN (none early), and three or more episodes of AGPN (none early). Thus, the time-dependent variable for AGPN allowed patients, initially all in the non-AGPN group, to change category at the time of a new episode. We included acute rejection and CMV infection post transplantation as time-dependent covariates in our cause-specific time-varying Cox models. For these two time-dependent covariates, the patients were considered exposed after their first episode of acute rejection or their first CMV infection.

To assess the effect of AGPN on the post transplantation evolution of the eGFR, we used a linear mixed effect model with random patient-specific intercepts and slopes. We compared the random intercept and slope model with a reduced model with only random intercepts using the Akaike information criterion; the full model suggested a better fit. We first estimated the relationship between the number of AGPN episodes at 3 months post transplantation and the evolution of the eGFR. We performed the same analysis for a baseline set at 6-, 12- and 24- months post transplantation. Because of the observed nonlinearity, we modeled the evolution of the eGFR according to two slopes before transplantation and 1-year post transplantation. Using baselines at 12- and 24-months post transplantation, we distinguished the number of AGPN episodes according to whether the first occurred early or late. For patients who returned to dialysis, we fixed the eGFR at 5 ml/min at the time of return to therapy. Residual analyses were performed to check the models’ validity.

For each outcome, we included the covariates that were significantly associated with the outcomes (p<0.05) in the univariate models in the multivariable models. We tested one time-dependent interaction term between the first rejection episode and the first AGPN episode in the Cox models and retained it if significant (p<0.05). In the eGRF analyses, we considered an interaction term between AGPN and the time from baseline to allow different evolutions of the eGFR in patients with AGPN. Patients with missing data for the covariates retained in the multivariable models or without measurements of the eGFR at baseline or subsequently were excluded. We described the characteristics of the studied patients and those of the excluded patients. We used R version 3.6.1 for all data analyses.

## Results

### Demographic Characteristics of Kidney Recipients

We included 9052 recipients of kidney (n= 8508) and combined kidney/pancreas (n= 544) transplants in this study. The characteristics of the patients stratified by type of AGPN episodes are described in **Table 1**. Among the 9052 included patients, 1766 (19.5%) experienced at least one AGPN episode during follow-up. Fifty-four percent of AGPN episodes occurred in the first 6 months after transplantation (early episodes of AGPN). The probabilities of having an AGPN episode at 1, 5- and 10-years post transplantation were 0.15 (95% confidence interval (CI) from 0.14 to 0.16), 0.24 (95% CI from 0.23 to 0.25) and 0.29 (95% CI from 0.28 to 0.31), respectively. Over the entire study period, the probabilities of rejection episodes and CMV infections were 22.3% and 14.5%, respectively. The median duration of follow-up for the 9052 recipients was 3.96 years (ranging from 0 to 12 years). During follow-up, 1072 patients returned to dialysis, 760 died with functioning grafts and 2431 patients developed *de novo* DSA.

### An Initial Episode of Late APGN Increases the Overall Risk of Graft Failure

**Figure 1:**
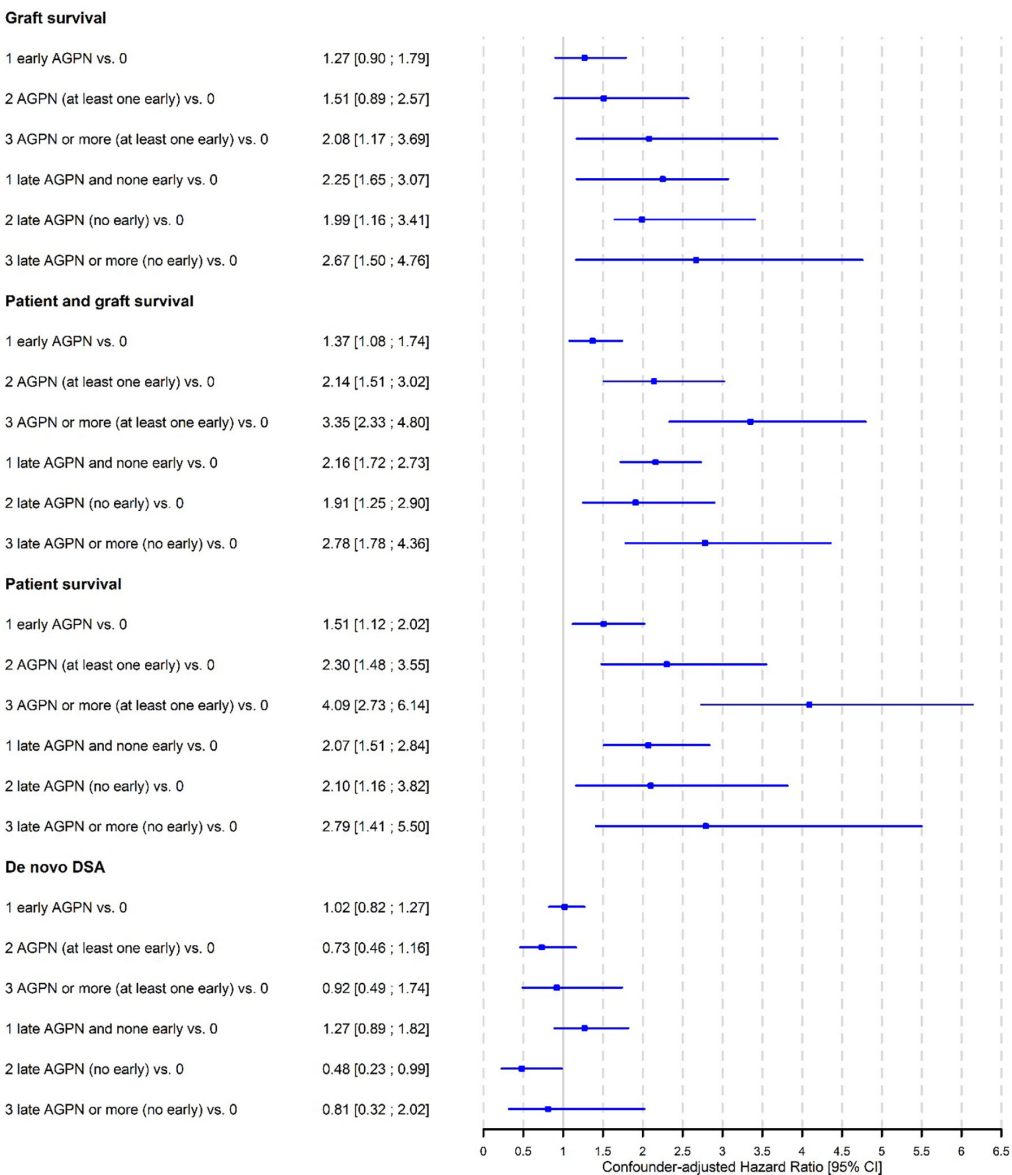
Confounder-adjusted Hazard Ratios according number of acute graft pyelonephritis (AGPN) episodes.

The cumulative incidence rates of graft failure at 5- and 10-years post transplantation were 12.6% (95% CI from 11.8% to 13.5%) and 25.4% (95% CI from 23.6% to 27.4%), respectively (**Figure S1**). **Table 2** and **Table S1** shows the unadjusted analyses and the final multivariable time-dependent Cox model for the risk of graft failure. The confounder-adjusted hazard ratios (HRs) associated with the number of AGPN episodes when the first AGPN episode occurred early were 1.27 for one AGPN episode (95% CI from 0.90 to 1.79), 1.51 for two AGPN episodes (95% CI from 0.89 to 2.57) and 2.08 for three or more AGPN episodes (95% CI from 1.17 to 3.69), each compared to no AGPN episodes. When the first AGPN episode occurred late, the HRs were 2.25 for one AGPN episode (95%CI from 1.65 to 3.07), 1.99 for two AGPN episodes (95%CI from 1.16 to 3.41) and 2.67 for three or more AGPN episodes (95%CI from 1.50 to 4.76), each compared to no AGPN episodes (**Figure 1**). Altogether, whereas we did not show a significant increase in the risk of graft failure until the third episode of AGPN when the first AGPN episode occurred early, we observed a significant two-fold higher risk of graft failure when the first episode of AGPN occurred late.

**Table 2:** Results of the unadjusted (univariate) and multivariable cause-specific time-dependent Cox models stratified on center studying the risk of graft failure.

	Unadjusted Cox models			Adjusted Cox model		
	HR	95% CI	p-value	HR	95% CI	p-value
**AGPN**			<0.0001			<0.0001
**One early (vs. None)**	1.57	[1.21; 2.02]		1.27	[0.90; 1.79]	
**Two at least the first occurs early (vs. None)**	1.96	[1.33; 2.90]		1.51	[0.89; 2.57]	
**Three or more at least the first occurs early (vs. None)**	2.12	[1.42; 3.17]		2.08	[1.17; 3.69]	
**One late AGPN and no early (vs. None)**	2.08	[1.62; 2.68]		2.25	[1.65; 3.07]	
**Two late AGPN and no early (vs. None)**	1.58	[0.97; 2.58]		1.99	[1.16; 3.41]	
**Three or more late AGPN and no early (vs. None)**	3.24	[2.15; 4.89]		2.67	[1.50; 4.76]	

AGPN, acute graft pyelonephritis; CI, confidence interval; HR, hazard ratio.

### A First Episode of Late AGPN Increases the Risk of Graft Failure or Death

The cumulative incidence rates of graft failure or death at 5- and 10-years post transplantation were 21.9% (95% CI from 20.8% to 23.0%) and 45.1% (95% CI from 42.8% to 47.4%), respectively (**Figure S1**). **Table 3** and **Table S2** shows the unadjusted analyses and the final multivariable Cox model. The confounder-adjusted HRs associated with the number of AGPN episodes when the first episode occurred early were 1.37 for one AGPN episode (95% CI from 1.08 to 1.74), 2.14 for two AGPN episodes (95% CI from 1.51 to 3.02) and 3.35 for three or more AGPN episodes (95% CI from 2.33 to 4.80), each compared to no AGPN episodes. These results indicate a significant increase in the risk of graft failure or death with a first early AGPN, with an increase in risk associated with subsequent episodes. When the first AGPN episode occurred late, the confounder-adjusted HRs were 2.16 for one AGPN episode (95% CI from 1.72 to 2.73), 1.91 for two AGPN episodes (95% CI from 1.25 to 2.90) and 2.78 for three or more AGPN episodes (95% CI from 1.78 to 4.36), each compared to no AGPN episodes. The risk increased two-fold with the first late episode and remained relatively stable with subsequent episodes. **Figure 1** summarizes these results.

**Table 3:** Results of the unadjusted (univariate) and multivariable cause-specific time-dependent Cox models stratified on center studying the risk of graft failure or death.

	Unadjusted Cox models			Adjusted Cox model		
	HR	95% CI	p-value	HR	95% CI	p-value
**AGPN**			<0.0001			<0.0001
**One early (vs. None)**	1.67	[1.40; 2.00]		1.37	[1.08; 1.74]	
**Two at least the first occurs early (vs. None)**	2.28	[1.74; 2.99]		2.14	[1.51; 3.02]	
**Three or more at least the first occurs early (vs. None)**	2.66	[2.02; 3.50]		3.35	[2.33; 4.80]	
**One late AGPN and no early (vs. None)**	2.11	[1.74; 2.55]		2.16	[1.72; 2.73]	
**Two late AGPN and no early (vs. None)**	1.81	[1.28; 2.57]		1.91	[1.25; 2.90]	
**Three or more late AGPN and no early (vs. None)**	2.89	[2.08; 4.02]		2.78	[1.78; 4.36]	

AGPN, acute graft pyelonephritis; CI, confidence interval; HR, hazard ratio.

### Patient Survival Decreases After an Early or Late First AGPN Episode

Cumulative rates of mortality with a functioning graft at 5- and 10-years post transplantation were 9.3% (95% CI from 8.5% to 10.1%) and 19.6% (95% CI from 18.0% to 21.4%), respectively (**Figure S1**). **Table 4** and **Table S3** presents the unadjusted analyses and the final multivariable Cox model. The confounder-adjusted HRs associated with the number of AGPN episodes when the first AGPN episode occurred early were 1.51 for one AGPN episode (95% CI from 1.12 to 2.02), 2.30 for two AGPN episodes (95% CI from 1.48 to 3.55) and 4.09 for three or more AGPN episodes (95% CI from 2.73 to 6.14), each compared to no AGPN episodes. When the first AGPN episode occurred late, they were 2.07 for one AGPN episode (95% CI from 1.51 to 2.84), 2.10 for two AGPN episodes (95% CI from 1.16 to 3.82) and 2.79 (95% CI from 1.41 to 5.50) for three or more AGPN episodes, each compared to no AGPN episodes (**Figure 1**). A first AGPN episode within 6 months post transplantation significantly increased patient mortality compared to no APGN episodes, and subsequent episodes of AGPNs after that first early episode negatively affected patient survival. Also, a late first AGPN episode is associated with a two-fold increase in the risk of patient mortality regardless of the number of subsequent episodes.

**Table 4:** Results of the unadjusted (univariate) and multivariable cause-specific time-dependent Cox models stratified on center studying the risk of death.

	Unadjusted Cox models			Adjusted Cox model		
	HR	95% CI	p-value	HR	95% CI	p-value
**AGPN**			<0.0001			<0.0001
**One early (vs. None)**	1.80	[1.40; 2.32]		1.51	[1.12; 2.02]	
**Two at least the first occurs early (vs. None)**	2.64	[1.83; 3.81]		2.30	[1.48; 3.55]	
**Three or more at least the first occurs early (vs. None)**	3.32	[2.29; 4.81]		4.09	[2.73; 6.14]	
**One late AGPN and no early (vs. None)**	2.13	[1.62; 2.81]		2.07	[1.51; 2.84]	
**Two late AGPN and no early (vs. None)**	2.10	[1.31; 3.37]		2.10	[1.16; 3.82]	
**Three or more late AGPN and no early (vs. None)**	2.45	[1.43; 4.22]		2.79	[1.41; 5.50]	

AGPN, acute graft pyelonephritis; CI, confidence interval; HR, hazard ratio.

### AGPN Has No Impact on the Risk of the *De Novo* Appearance of DSA

A total of 7353 patients were included in the analysis of the *de novo* appearance of DSA, and 1699 were excluded due to missing data on DSA post transplantation. In the multivariable analyses, 1799 supplementary patients were excluded due to missing data for covariates. The cumulative incidence rates of the *de novo* appearance of DSA at 5- and 10-years post transplantation were 38.0% (95% CI from 36.7% to 39.4%) and 55.3% (95% CI from 52.8% to 57.8%), respectively (**Figure S2**). **Table 5** and **Table S4** presents the unadjusted analyses and the final multivariable Cox model. Altogether, we found no significant association between the timing or the number of AGPN episodes and the risk of the *de novo* appearance of DSA (*P* = 0.2474).

**Table 5:** Results of the unadjusted (univariate) and multivariable cause-specific time-dependent Cox models stratified on center studying the risk of occurrence of *de novo* DSA.

	Unadjusted Cox models			Adjusted Cox model		
	HR	95% CI	p-value	HR	95% CI	p-value
**AGPN**			0.5702			0.2474
**One early (vs. None)**	1.14	[0.96; 1.36]		1.02	[0.82; 1.27]	
**Two at least the first occurs early (vs. None)**	1.15	[0.84; 1.57]		0.73	[0.46; 1.16]	
**Three or more at least the first occurs early (vs. None)**	0.87	[0.53; 1.44]		0.92	[0.49; 1.74]	
**One late AGPN and no early (vs. None)**	1.15	[0.87; 1.52]		1.27	[0.89; 1.82]	
**Two late AGPN and no early (vs. None)**	0.82	[0.50; 1.37]		0.48	[0.23; 0.99]	
**Three or more late AGPN and no early (vs. None)**	1.08	[0.58; 2.02]		0.81	[0.32; 2.02]	

AGPN, acute graft pyelonephritis; CI, confidence interval; HR, hazard ratio.

### Early AGPN Episodes Are Associated With a Lower eGFR

Among the 9052 kidney recipients, 7992 patients had at least one measurement of the eGFR at 3 months post transplantation or later. Among them, 588 had one AGPN episode, and 106 had 2 or more AGPN episodes in the first 3 months post transplantation. The mean number of eGFR assessments was 5.7 (range from 1 to 14) after a mean of 29.8 months (range from 1 to 147). The mean 3-month eGFRs were 51.9 ml/min (95% CI from 51.4 to 52.3) for patients without any AGPN episodes at 3 months, 47.1 ml/min (95% CI from 45.5 to 48.8) for patients with one AGPN episode at 3 months and 45.3 ml/min (95% CI from 41.5 to 49.2) for patients with 2 or more AGPN episodes at 3 months. A total of 724 recipients returned to dialysis during follow-up. **Table 6** presents the results of the multivariable linear mixed model. We observed that the confounder-adjusted mean eGFR at 3 months was significantly lower for recipients with one AGPN episode (- 2.6 ml/min, 95% CI from - 4.2 to - 1.0) or two or more AGPN episodes (-4.5 ml/min, 95% CI from – 8.1 to - 0.9) than for patients without any AGPN episodes (**Table 6** and **Table S5**). Overall, we estimated an increase of 0.07 ml/min per month (95% CI from 0.04 to 0.10) between 3- and 12- months post transplantation and then a decrease of 0.11 ml/min per month thereafter (95% CI from 0.09 to 0.12). However, this evolution of the eGFR was not significantly different among the three groups (no AGPN episodes, one AGPN episode and two or more AGPN episodes). The result at 6 months post transplantation was similar (**Table S1**).

**Table 6:** Results of the multivariable linear mixed model of eGFR (ml/min/1.73m^2^) including random intercept and random slope when the baseline was set at 3 months post-transplantation.

	3 months post-transplantation		
	Adjusted mean difference	95% CI	p-value
**AGPN**			0.0004
**One early (vs. None)**	-2.62	[-4.22; -1.02]	
**Two or more (vs. None)**	-4.49	[-8.07; -0.91]	

AGPN, acute graft pyelonephritis; CI, confidence interval

Therefore, although we did not observe a difference in the evolution of the eGFR, the levels of renal function remained significantly lower at 3- and 6-months post transplantation for patients with early AGPN episodes.

**Figure 2:**
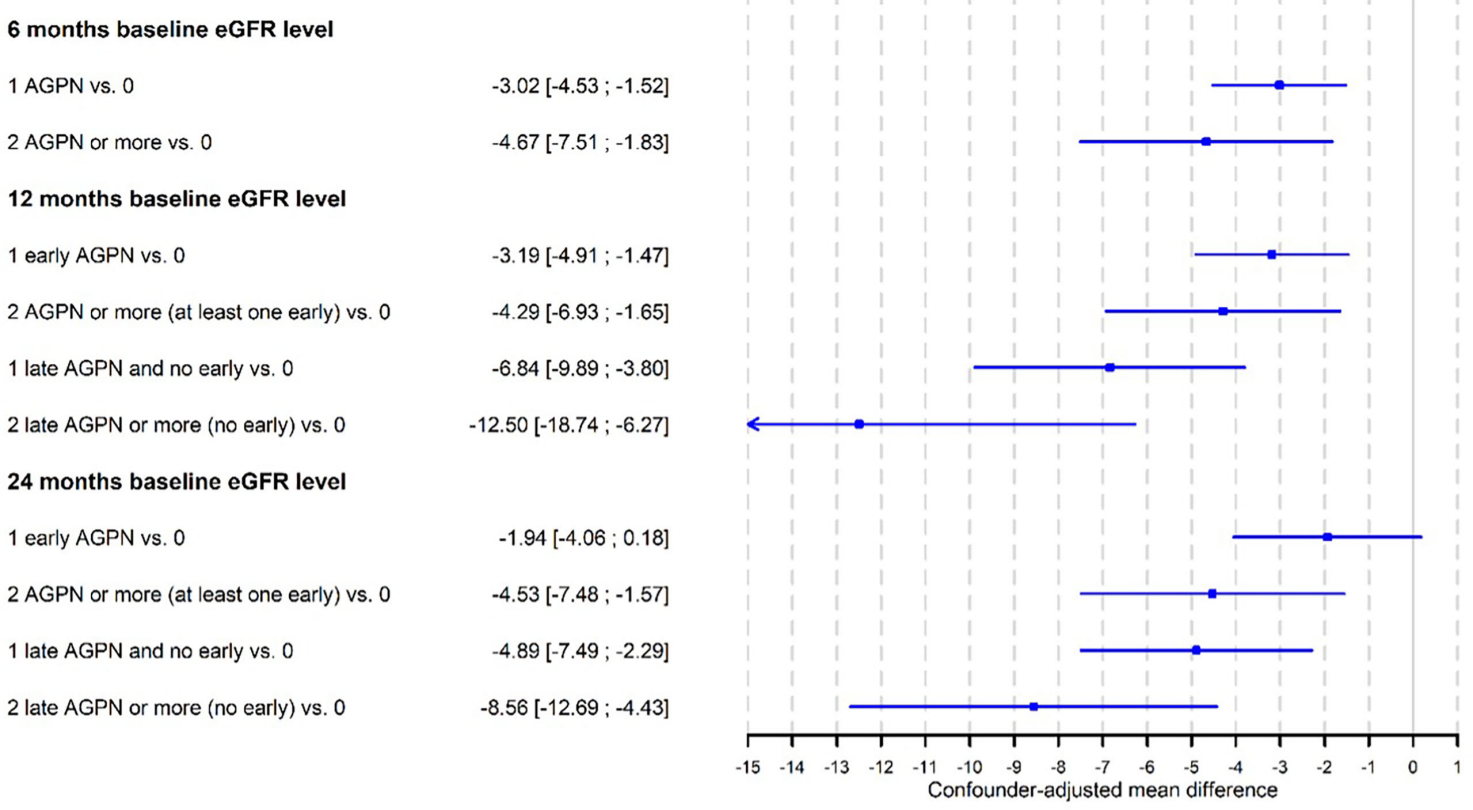
Confounder-adjusted mean differences of eGFR at baseline according to the number of acute graft pyelonephritis (AGPN) episodes estimated from multivariable linear-mixed models.

For the baseline sets at 12- and 24-months post transplantation, we distinguished the number of episodes according to whether the first AGPN episode occurred within 6 months after transplantation or later (**Tables S6–S7**). We observed a more important decrease in the level of the eGFR at 12 months post transplantation when the first AGPN episode was late (- 6.8 ml/min, 95% CI from – 9.9 to – 3.8) compared to no AGPN episodes than when the first AGPN episode was early (- 3.2 ml/min, 95% CI from - 4.9 to – 1.7) compared to no AGPN episodes. In addition, the results at 24 months post transplantation remained unchanged. The confounder-adjusted mean differences in the eGFR at baseline according to the number of AGPN episodes are summarized in **Figure 2**. Altogether, late AGPN episodes reduce the eGFR to a significantly greater extent than early AGPN episodes compared to no AGPN episodes.

## Discussion

It is largely admitted that urinary tracts infections have a deleterious impact on long term graft survival in kidney transplanted recipients (2, 4, 6). In our study, we could individualize the negative impact of early and late episode of AGPN on graft survival, patient survival and eGFR. In our multicentric cohort of 9052 transplant recipients, we found an incident rate of AGPN of 20.9% in accordance with previous cohorts (6, 8, 9). Of note, 54% of APGN episodes occurred within the first 6 months after transplantation suggesting a role of the location of the surgical procedure, the presence of the ureteral stents (18–20) and the use of anti-thymoglobulin (19). Furthermore, previous reports on small cohorts suggested an increased risk of post-transplant urinary infections in recipients with recurrent UITs or vesicoureteral reflux prior to kidney transplantation (21, 22). Due to our database coding, their respective impact could not be determined.

We reported that a first episode of late AGPN (i.e., after 6 months post transplantation) was associated with a twice higher and independent risk of both mortality and long-term graft failure than in patients with no AGPN episodes. To our knowledge, only one study focused on the confounder-adjusted impact of late AGPN after kidney transplantation. Abbott and al, performed a retrospective cohort study on 28 942 kidney transplanted recipients from 1996 to 2000 in the United States Renal Data System. Thanks to an equivalent method, the authors found an increased risk of death (HR = 2.93 (2.22 – 3.85)) and graft loss (HR = 1.85 (1.29 – 2.64)) when UTI occurred 6 months after transplantation (10). Despite scare data, those data strongly suggest a negative impact of a first episode of late AGPN on graft and patient survival.

An original aspect of our work is that we estimated the effect of both repeated AGPN episodes according to their onset (early versus late). We reported that patient death, graft failure and combined patient-graft death were significantly increased in case of repeated AGPN episodes, especially when the first episode occurred early within the first 6 months after transplantation. The impact of recurrent UTI in kidney transplanted recipients has been poorly explored. Two recent retrospective cohort studies also associated recurrent UTIs with a twice higher risk of graft loss all the more so one episode of UTI occurred within the first year after transplantation (23, 24). Of note, there was no distinction between early and late onset of UTI. Interestingly, we observed that a single episode of late AGPN was necessary to reach the same risk of graft failure and/or patient death in our cohort questioning about how post-transplantation time to AGPN onset can impact graft function. Dupont and al, described several patterns of multiple cortical scarring of the graft thanks to photo emission computed tomography. Those patterns were strongly associated with a long-term decreased graft function (25). In the same line, Cartery and al performed systematic graft biopsies one month after an episode of AGPN and compared them to protocol kidney biopsy at one year that did not present AGPN. The authors observed more frequent tubulitis histologic patterns after AGPN all the more so eGFR did not return to baseline (26), which makes an echo to our data where AGPN were associated with a lower eGFR without altering significantly eGFR decay. The authors also observed an increased CNI through level in the AGPN group (26). Altogether, those data argued that recurrent AGPN after kidney transplantation were associated with a poorer graft function but also a poorer patient survival all the more so the first recurrent episode occurred early with in the first six months post-transplant. This effect could be explained by multiple residual scarring. High CNI through level and their pro-fibrosis effect might take part and would partly explain why a single episode of late AGPN (i.e., after 6 months post-transplant) was associated to the same poor graft-patient survival. Unfortunately, we could explore this hypothesis due to 4/5^th^ missing data to calculate CNI through level during follow-up in our cohort. At last, the proportion of relevant urological medical history (nephrectomy, surgical procedures on urinary tract for stenosis, reflux or malformative uropathy) in our cohort at baseline was significantly higher in recipients with AGPN than recipients without AGPN (early and late) during follow-up (p-value = 4x10- (12); Chi^2^). No difference was observed between recipients with early and late AGPN (p-value = 0.9; Chi^2^). Dupont et al, also highlighted that vesicoureteral reflux but not exclusively was associated with cortical scarring and poor long-term graft function (25). Combining these data, we hypothesized that kidney transplanted recipients with relevant urological history would be likely develop recurrent or late onset AGPN leading to poorer graft function and increased patient death.

In our cohort, we did not find neither any interaction between AGPN onset and acute allograft rejection nor any association with the outcomes of our study (**Tables S1, S2, S4**). When it comes to treatments used for acute allograft rejection, we could observe a higher proportion of oral corticoids and humoral modulators (plasmapheresis, IVIg and rituximab) in the AGPN groups compared to no AGPN group. Though, no group comparison was performed to avoid immortal time bias (27). In a previous cohort of kidney transplanted recipient, our group demonstrated that AGPN occurred more likely 1) after acute rejection and 2) after CMV infection (6). This suggested that AGPN would be a consequence of over immunosuppression induced by acute rejection treatments, which was concordant with the former observations in our cohort. Therefore, treatment rejections might appear as up-stream mediators of AGPN onset during follow-up after kidney transplantation than confounding factors on allograft failure, patient death or baseline eGFR decrease.

Somehow, our observational study suffers from limitations. First, the AGPN group was heterogeneous, with some UTIs that could increase the risk of mortality and graft failure. Second, we did not identify the microorganisms responsible for AGPN. Some microorganisms, in particular *Escherichia coli*, could have a larger impact on the fate of grafts (28). The importance of infections with multidrug-resistant microorganisms, which can contribute to difficulties with antibiotic treatment and favor recurrence, has not yet been evaluated (25, 29). Third, the impact of CNI through level as a confounding factor on our outcomes after AGPN during follow-up could not be evaluated due to missing data. At last, ethnicity and socioeconomic status were not collected due to French law regulations though their assessment might be important to more accurately estimate the eGFR. Despite those limitations, the multicentric design, the prospective data collection the DIVAT cohort and the number of patients included in our study argue for a generalizability of our results to the current population of kidney transplanted recipients.

To conclude, we showed that a single episode of late AGPN occurring after 6 months post transplantation or recurrent episodes of AGPN with the first occurring within 6 months post-transplantation were associated with a twice a higher risk of graft failure, patient death and eGFR decay. Thus, AGPN after kidney transplantation should not be considered a benign infection from the first episode. Although risk factors for early or late onset AGPN episode still remain unclear, our results therefore emphasize the need to monitor closely eGFR evolution, to explore vesicoureteral reflux, to provide medical advice to minimize the risk of recurrence (cranberry extract daily intake, daily water intake > 1,5L/day and regular urination across the day) after a single episode of APGN in kidney transplanted recipients. Low dose long-term antibiotics could be discussed bearing in mind that multidrug resistant bacteria in the urine of kidney transplanted recipients could be a risk factor for AGPN (25, 29). Additional studies should be conducted to evaluate/confirm the risk factors for the occurrence of late episodes of AGPN in clinical practice.

## Data Availability Statement

The original contributions presented in the study are included in the article/**Supplementary Material**. Further inquiries can be directed to the corresponding author.

## DIVAT Cohort Collaborators (Medical Doctors, Surgeons, HLA Biologists)

Nantes: Gilles Blancho, Julien Branchereau, Diego Cantarovich, Agnès Chapelet, Clément Deltombe, Lucile Figueres, Claire Garandeau, Magali Giral, Caroline Gourraud-Vercel, Maryvonne Hourmant, Georges Karam, Clarisse Kerleau, Aurélie Meurette, Simon Ville, Christine Kandell, Anne Moreau, Karine Renaudin, Anne Cesbron, Florent Delbos, Alexandre Walencik, Anne Devis; Paris-Necker: Lucile Amrouche, Dany Anglicheau, Olivier Aubert, Lynda Bererhi, Christophe Legendre, Alexandre Loupy, Frank Martinez, Rébecca Sberro-Soussan, Anne Scemla, Claire Tinel, Julien Zuber.

## Author Contributions

MP: Conceptualization, Methodology, Formal analysis, Writing - original draft. LC: Methodology, Writing - original draft, review and editing. JD: Conceptualization, Methodology, Writing - review and editing. SB: Conceptualization, Methodology, Writing - review and editing. CK: data management. FB: Methodology, Statistical analysis, Review and editing. MG: Data acquisition, Writing. All authors contributed to the article and approved the submitted version.

## Funding

This work was supported by the CENTAURE foundation (www.fondation-centaure.org), which supports a French transplantation research network, the IHU-Cesti project, the DHU Oncogreffe and the LabEX IGO courtesy of French government financial support managed by the National Research Agency *via* the “Investment into the Future” program (ANR-10-IBHU-005 and ANR-11- LABX-0016-01). The IHU-Cesti project is also supported by Nantes Métropole and Région Pays de la Loire. This work was performed in the context of DHU Oncogreffe, LabEX IGO (ANR-11- LABX-0016-01), ANR project BIKET (ANR-17-CE17-0008) and ANR project KTD-innov (ANR-17-RHUS-0010) courtesy of French government financial support managed by the National Research Agency. The laboratory received funding from the European Union’s Horizon 2020 Research and Innovation Programme under Grant Agreement No. 754995.

## Conflict of Interest

FB is employed by IDBC-A2COM.

The remaining authors declare that the research was conducted in the absence of any commercial or financial relationships that could be construed as a potential conflict of interest.

## Publisher’s Note

All claims expressed in this article are solely those of the authors and do not necessarily represent those of their affiliated organizations, or those of the publisher, the editors and the reviewers. Any product that may be evaluated in this article, or claim that may be made by its manufacturer, is not guaranteed or endorsed by the publisher.
