# Supplementary material for: Impact of Late and Recurrent Acute Graft Pyelonephritis on Long-Term Kidney Graft Outcomes
Source: Front Immunol. 2022 Mar 2;13:824425. doi: 10.3389/fimmu.2022.824425 (PMC8998071; doi:10.3389/fimmu.2022.824425)

Figure S1. Cumulative incidence curves estimated with the Aalen Johansen estimator of graft failure (deaths as competing events), graft failure or death, death with functioning graft (return to dialysis or pre-emptive re-transplantations as competing events).


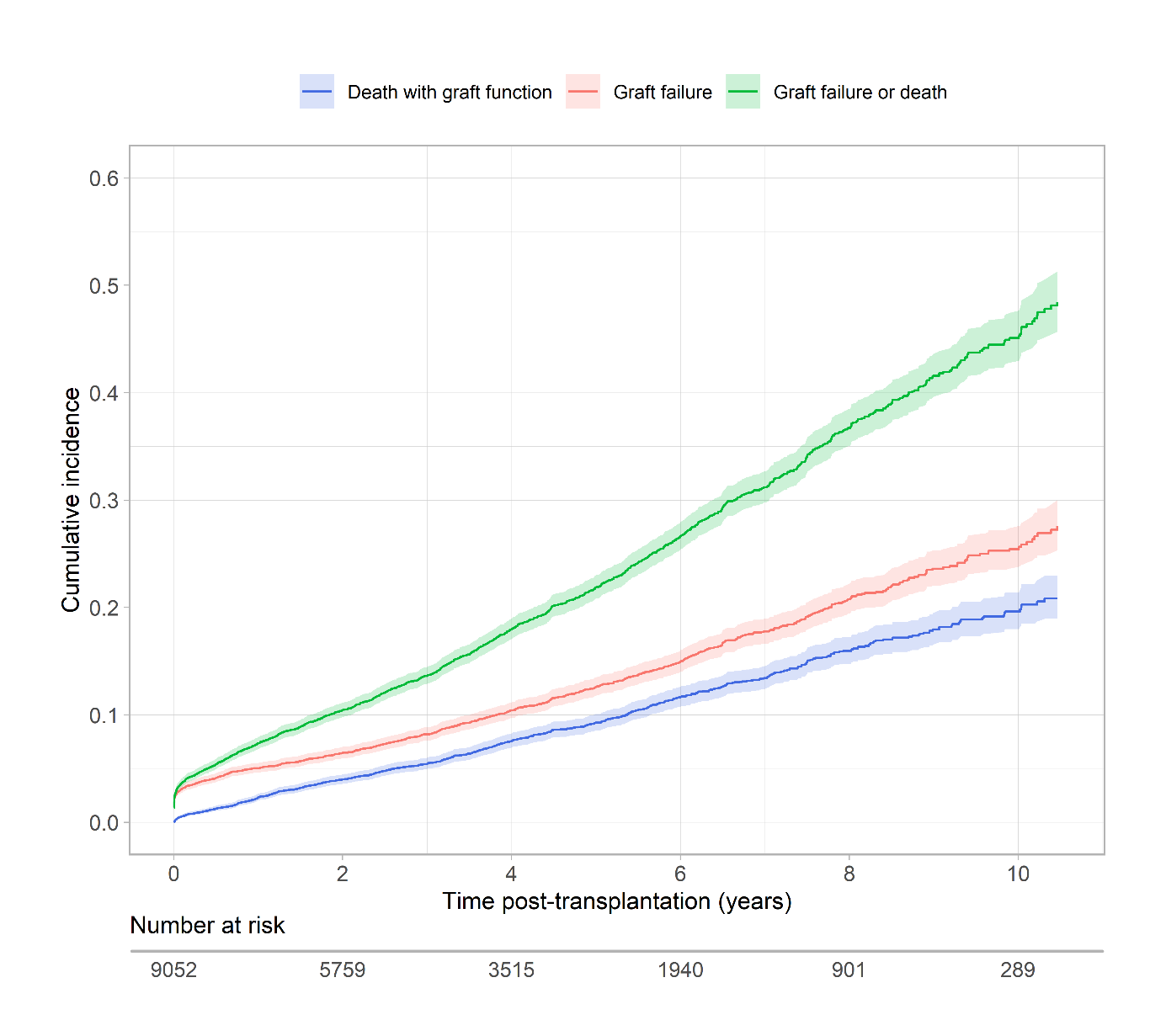

Supplement: Supplementary file 1 [file DataSheet_1.docx]
