# Supplementary material for: Impact of Late and Recurrent Acute Graft Pyelonephritis on Long-Term Kidney Graft Outcomes
Source: Front Immunol. 2022 Mar 2;13:824425. doi: 10.3389/fimmu.2022.824425 (PMC8998071; doi:10.3389/fimmu.2022.824425)

Figure S2. Cumulative incidence curve of occurrence of de novo DSA (Aalen-Johansen estimator, re-transplantations, returns to dialysis and deaths as competing events).


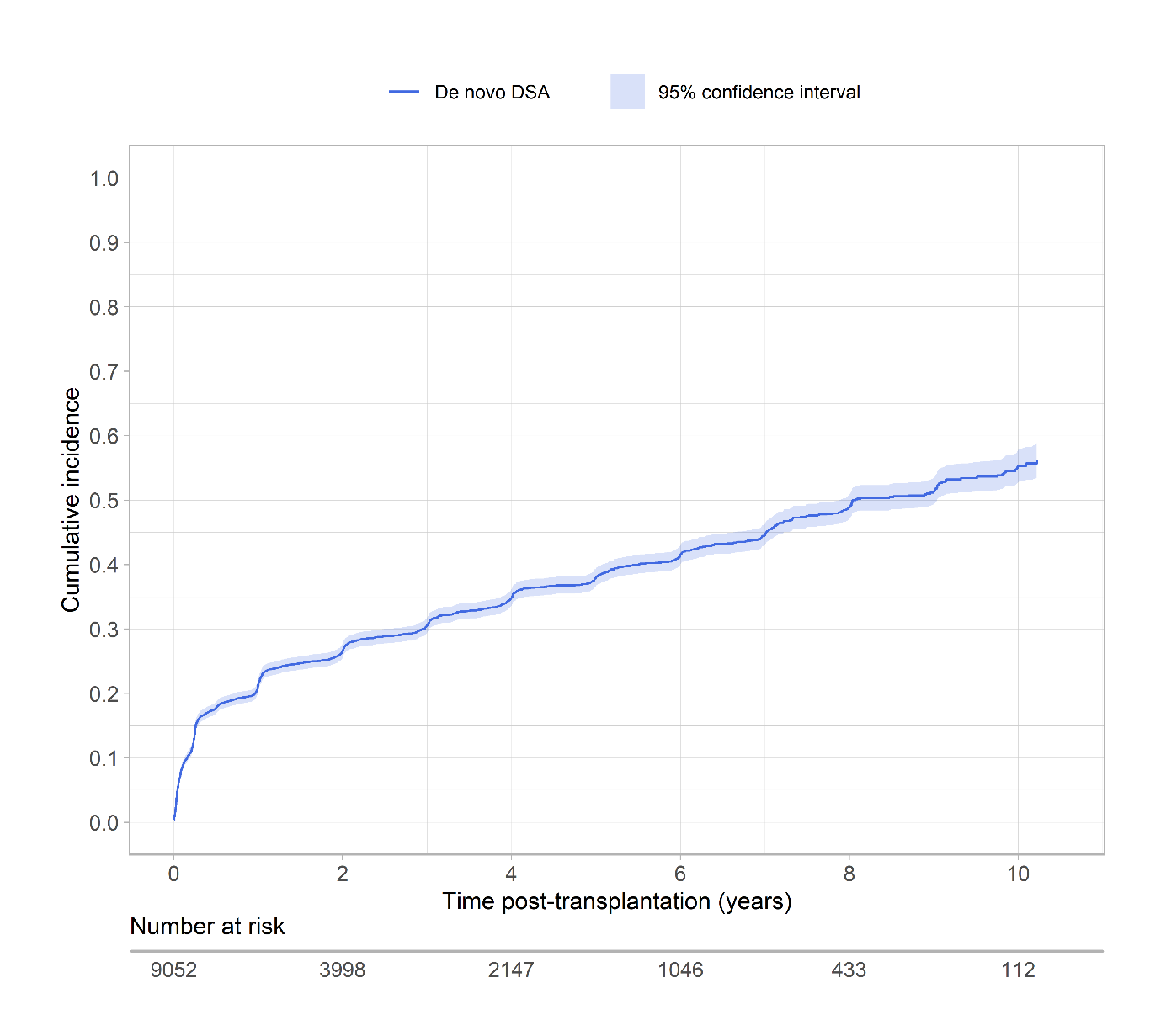

Supplement: Supplementary file 2 [file DataSheet_2.docx]
