# Supplementary material for: Impact of Late and Recurrent Acute Graft Pyelonephritis on Long-Term Kidney Graft Outcomes
Source: Front Immunol. 2022 Mar 2;13:824425. doi: 10.3389/fimmu.2022.824425 (PMC8998071; doi:10.3389/fimmu.2022.824425)
Supplement: Supplementary file 3 [file Table_1.docx]

**Table S1.** Results of the unadjusted and multivariable cause-specific time-dependent Cox models stratified on center studying the risk of graft failure.

|  | **Unadjusted Cox models** | | | **Adjusted Cox model** | | |
| --- | --- | --- | --- | --- | --- | --- |
|  | **HR** | **95% CI** | **p-value** | **HR** | **95% CI** | **p-value** |
| **AGPN** |  |  | <0.0001 |  |  | <0.0001 |
| **One early (vs. None)** | 1.57 | [1.21 ; 2.02] |  | 1.27 | [0.90 ; 1.79] |  |
| **Two at least the first occurs early (vs. None)** | 1.96 | [1.33 ; 2.90] |  | 1.51 | [0.89 ; 2.57] |  |
| **Three or more at least the first occurs early (vs. None)** | 2.12 | [1.42 ; 3.17] |  | 2.08 | [1.17 ; 3.69] |  |
| **One late AGPN and no early (vs. None)** | 2.08 | [1.62 ; 2.68] |  | 2.25 | [1.65 ; 3.07] |  |
| **Two late AGPN and no early (vs. None)** | 1.58 | [0.97 ; 2.58] |  | 1.99 | [1.16 ; 3.41] |  |
| **Three or more late AGPN and no early (vs. None)** | 3.24 | [2.15 ; 4.89] |  | 2.67 | [1.50 ; 4.76] |  |
| **Recipient age** ≥**55 years** | 1.25 | [1.10 ; 1.43] | 0.0006 | 1.06 | [0.86 ; 1.29] | 0.5904 |
| **Retransplantation** | 1.30 | [1.11 ; 1.53] | 0.0013 | 1.13 | [0.87 ; 1.45] | 0.3602 |
| **Male sex** | 1.00 | [0.88 ; 1.14] | 0.9734 | - | - |  |
| **Kidney transplantation (vs SPK transplantation)** | 1.01 | [0.75 ; 1.36] | 0.9424 | - | - |  |
| **Renal replacement therapy** |  |  | <0.0001 |  |  | 0.9285 |
| - **Hemodialysis (vs. Preemptive transplantation)** | 1.80 | [1.46 ; 2.21] |  | 1.05 | [0.79 ; 1.39] |  |
| - **Peritoneal dialysis (vs. Preemptive transplantation)** | 1.45 | [1.08 ; 1.95] |  | 1.08 | [0.73 ; 1.59] |  |
| **History of diabetes** | 1.36 | [1.17 ; 1.57] | <0.0001 | 1.29 | [1.05 ; 1.57] | 0.0131 |
| **Recurrent initial nephropathy** | 1.13 | [0.98 ; 1.30] | 0.0933 | - | - |  |
| **Uro-nephrological history** | 0.97 | [0.79 ; 1.18] | 0.7338 | - | - |  |
| **Positive recipient CMV serology** | 1.08 | [0.94 ; 1.24] | 0.2908 | - | - |  |
| **Deceased donor** | 2.22 | [1.77 ; 2.78] | <0.0001 | 1.55 | [1.15 ; 2.09] | 0.0037 |
| **Donor age** ≥**55 years** | 1.55 | [1.36 ; 1.76] | <0.0001 | 1.53 | [1.25 ; 1.88] | <0.0001 |
| **Male donor** | 0.95 | [0.83 ; 1.08] | 0.4215 | - | - |  |
| **Positive donor CMV serology** | 1.06 | [0.93 ; 1.20] | 0.4122 | - | - |  |
| **Cold ischemia time** ≥**18 hours** | 1.34 | [1.17 ; 1.53] | <0.0001 | 0.93 | [0.76 ; 1.13] | 0.4617 |
| **Depleting induction at transplantation** | 1.25 | [1.07 ; 1.45] | 0.0037 | 1.21 | [0.98 ; 1.48] | 0.0729 |
| **Maintenance CNI treatment at transplantation** |  |  | <0.0001 |  |  | 0.2943 |
| - **Ciclosporine (vs. Tacrolimus)** | 1.23 | [1.03 ; 1.46] |  | 0.88 | [0.67 ; 1.16] |  |
| - **None (vs. Tacrolimus)** | 2.43 | [1.65 ; 3.60] |  | 1.56 | [0.76 ; 3.19] |  |
| **CMV prophylaxis treatment at transplantation** | 0.95 | [0.80 ; 1.12] | 0.5142 | - | - |  |
| **Immunization pre-transplantation** |  |  | 0.0085 |  |  | 0.5782 |
| - **anti-HLA no DSA (vs. Negative)** | 1.15 | [0.98 ; 1.35] |  | 1.11 | [0.89 ; 1.39] |  |
| - **DSA (vs. Negative)** | 1.48 | [1.15 ; 1.91] |  | 1.17 | [0.83 ; 1.65] |  |
| **HLA-A-B-DR mismatches > 4** | 1.25 | [1.06 ; 1.46] | 0.0065 | 1.24 | [1.00 ; 1.53] | 0.0476 |
| **Pregnancy history** | 0.77 | [0.63 ; 0.94] | 0.0089 | 0.59 | [0.46 ; 0.75] | <0.0001 |
| **Delayed graft function** | 2.02 | [1.74 ; 2.35] | <0.0001 | 1.60 | [1.31 ; 1.96] | <0.0001 |
| **At least one rejection** | 4.33 | [3.72 ; 5.05] | <0.0001 | 3.98 | [3.26 ; 4.86] | <0.0001 |
| **At least one rejection x At least one AGPN** |  |  |  | 0.81 | [0.54 ; 1.20] | 0.2884 |
| **At least one CMV infection** | 1.73 | [1.44 ; 2.08] | <0.0001 | 1.44 | [1.13 ; 1.82] | 0.0028 |
| AGPN, acute graft pyelonephritis, CI, confidence interval; CMV, cytomegalovirus; DSA, donor-specific antibodies; HLA, human leucocyte antigens; HR, hazard ratio. | | | | | | |

**Table S2.** Results of the unadjusted and multivariable cause-specific time-dependent Cox models stratified on center studying the risk of graft failure or death.

|  | **Unadjusted Cox models** | | | **Adjusted Cox model** | | |
| --- | --- | --- | --- | --- | --- | --- |
|  | **HR** | **95% CI** | **p-value** | **HR** | **95% CI** | **p-value** |
| **AGPN** |  |  | <0.0001 |  |  | <0.0001 |
| **One early (vs. None)** | 1.67 | [1.40 ; 2.00] |  | 1.37 | [1.08 ; 1.74] |  |
| **Two at least the first occurs early (vs. None)** | 2.28 | [1.74 ; 2.99] |  | 2.14 | [1.51 ; 3.02] |  |
| **Three or more at least the first occurs early (vs. None)** | 2.66 | [2.02 ; 3.50] |  | 3.35 | [2.33 ; 4.80] |  |
| **One late AGPN and no early (vs. None)** | 2.11 | [1.74 ; 2.55] |  | 2.16 | [1.72 ; 2.73] |  |
| **Two late AGPN and no early (vs. None)** | 1.81 | [1.28 ; 2.57] |  | 1.91 | [1.25 ; 2.90] |  |
| **Three or more late AGPN and no early (vs. None)** | 2.89 | [2.08 ; 4.02] |  | 2.78 | [1.78 ; 4.36] |  |
| **Recipient age** ≥**55 years** | 2.05 | [1.87 ; 2.26] | <0.0001 | 1.80 | [1.55 ; 2.10] | <0.0001 |
| **Retransplantation** | 1.29 | [1.15 ; 1.45] | <0.0001 | 1.15 | [0.96 ; 1.39] | 0.1379 |
| **Male sex** | 1.10 | [1.00 ; 1.21] | 0.0547 | - | - |  |
| **Kidney transplantation (vs SPK transplantation)** | 1.23 | [0.97 ; 1.56] | 0.0838 | - | - |  |
| **Renal replacement therapy** |  |  | <0.0001 |  |  | 0.0377 |
| - **Hemodialysis (vs. Preemptive transplantation)** | 2.12 | [1.81 ; 2.49] |  | 1.34 | [1.07 ; 1.68] |  |
| - **Peritoneal dialysis (vs. Preemptive transplantation)** | 1.52 | [1.21 ; 1.91] |  | 1.33 | [0.98 ; 1.80] |  |
| **History of diabetes** | 1.65 | [1.49 ; 1.83] | <0.0001 | 1.61 | [1.40 ; 1.85] | <0.0001 |
| **Recurrent initial nephropathy** | 0.90 | [0.81 ; 1.01] | 0.0679 | - | - |  |
| **Uro-nephrological history** | 0.99 | [0.86 ; 1.15] | 0.9399 | - | - |  |
| **Positive recipient CMV serology** | 1.12 | [1.02 ; 1.24] | 0.0240 | 0.87 | [0.75 ; 0.99] | 0.0402 |
| **Deceased donor** | 2.34 | [1.99 ; 2.76] | <0.0001 | 1.55 | [1.24 ; 1.95] | 0.0001 |
| **Donor age** ≥**55 years** | 1.91 | [1.74 ; 2.10] | <0.0001 | 1.32 | [1.13 ; 1.53] | 0.0005 |
| **Male donor** | 0.95 | [0.86 ; 1.04] | 0.2505 | - | - |  |
| **Positive donor CMV serology** | 1.16 | [1.06 ; 1.27] | 0.0020 | 1.15 | [1.01 ; 1.32] | 0.0350 |
| **Cold ischemia time** ≥**18 hours** | 1.38 | [1.25 ; 1.52] | <0.0001 | 0.95 | [0.82 ; 1.09] | 0.4511 |
| **Depleting induction at transplantation** | 1.15 | [1.04 ; 1.28] | 0.0077 | 1.11 | [0.95 ; 1.29] | 0.1764 |
| **Maintenance CNI treatment at transplantation** |  |  | <0.0001 |  |  | 0.1105 |
| - **Ciclosporine (vs. Tacrolimus)** | 1.17 | [1.02 ; 1.33] |  | 0.92 | [0.76 ; 1.11] |  |
| - **None (vs. Tacrolimus)** | 2.80 | [2.10 ; 3.73] |  | 1.65 | [0.98 ; 2.78] |  |
| **CMV prophylaxis treatment at transplantation** | 0.92 | [0.81 ; 1.04] | 0.1730 | - | - |  |
| **Immunization pre-transplantation** |  |  | 0.0241 |  |  | 0.5402 |
| - **anti-HLA no DSA (vs. Negative)** | 1.13 | [1.01 ; 1.27] |  | 1.09 | [0.93 ; 1.29] |  |
| - **DSA (vs. Negative)** | 1.25 | [1.05 ; 1.49] |  | 1.09 | [0.86 ; 1.38] |  |
| **HLA-A-B-DR mismatches > 4** | 1.09 | [0.97 ; 1.23] | 0.1401 | - | - |  |
| **Pregnancy history** | 0.81 | [0.71 ; 0.93] | 0.0030 | 0.58 | [0.48 ; 0.70] | <0.0001 |
| **Delayed graft function** | 1.99 | [1.79 ; 2.22] | <0.0001 | 1.46 | [1.27 ; 1.69] | <0.0001 |
| **At least one rejection** | 2.42 | [2.16 ; 2.71] | <0.0001 | 2.12 | [1.82 ; 2.46] | <0.0001 |
| **At least one rejection x At least one AGPN** |  |  |  | 0.81 | [0.60 ; 1.08] | 0.1550 |
| **At least one CMV infection** | 1.60 | [1.40 ; 1.84] | <0.0001 | 1.21 | [1.01 ; 1.46] | 0.0391 |
| AGPN, acute graft pyelonephritis, CI, confidence interval; CMV, cytomegalovirus; DSA, donor-specific antibodies; HLA, human leucocyte antigens; HR, hazard ratio. | | | | | | |

**Table S3.** Results of the unadjusted and multivariable cause-specific time-dependent Cox models stratified on center studying the risk of death.

|  | **Unadjusted Cox models** | | | **Adjusted Cox model** | | |
| --- | --- | --- | --- | --- | --- | --- |
|  | **HR** | **95% CI** | **p-value** | **HR** | **95% CI** | **p-value** |
| **AGPN** |  |  | <0.0001 |  |  | <0.0001 |
| **One early (vs. None)** | 1.80 | [1.40 ; 2.32] |  | 1.51 | [1.12 ; 2.02] |  |
| **Two at least the first occurs early (vs. None)** | 2.64 | [1.83 ; 3.81] |  | 2.30 | [1.48 ; 3.55] |  |
| **Three or more at least the first occurs early (vs. None)** | 3.32 | [2.29 ; 4.81] |  | 4.09 | [2.73 ; 6.14] |  |
| **One late AGPN and no early (vs. None)** | 2.13 | [1.62 ; 2.81] |  | 2.07 | [1.51 ; 2.84] |  |
| **Two late AGPN and no early (vs. None)** | 2.10 | [1.31 ; 3.37] |  | 2.10 | [1.16 ; 3.82] |  |
| **Three or more late AGPN and no early (vs. None)** | 2.45 | [1.43 ; 4.22] |  | 2.79 | [1.41 ; 5.50] |  |
| **Recipient age** ≥**55 years** | 4.03 | [3.44 ; 4.72] | <0.0001 | 3.06 | [2.42 ; 3.86] | <0.0001 |
| **Retransplantation** | 1.29 | [1.08 ; 1.55] | 0.0054 | 1.21 | [0.96 ; 1.51] | 0.1070 |
| **Male sex** | 1.29 | [1.11 ; 1.50] | 0.0012 | 1.39 | [0.93 ; 2.09] | 0.1123 |
| **Kidney transplantation (vs SPK transplantation)** | 1.56 | [1.02 ; 2.39] | 0.0400 | 0.98 | [0.55 ; 1.75] | 0.9416 |
| **Renal replacement therapy** |  |  | <0.0001 |  |  | 0.0039 |
| - **Hemodialysis (vs. Preemptive transplantation)** | 2.72 | [2.07 ; 3.55] |  | 1.79 | [1.27 ; 2.52] |  |
| - **Peritoneal dialysis (vs. Preemptive transplantation)** | 1.77 | [1.22 ; 2.58] |  | 1.57 | [1.00 ; 2.46] |  |
| **History of diabetes** | 2.16 | [1.86 ; 2.52] | <0.0001 | 1.78 | [1.47 ; 2.15] | <0.0001 |
| **Recurrent initial nephropathy** | 0.67 | [0.56 ; 0.80] | <0.0001 | 0.87 | [0.70 ; 1.08] | 0.1996 |
| **Uro-nephrological history** | 0.97 | [0.77 ; 1.22] | 0.8164 | - | - |  |
| **Positive recipient CMV serology** | 1.14 | [0.98 ; 1.33] | 0.0957 | - | - |  |
| **Deceased donor** | 2.75 | [2.11 ; 3.59] | <0.0001 | 1.65 | [1.18 ; 2.30] | 0.0037 |
| **Donor age** ≥**55 years** | 2.43 | [2.10 ; 2.83] | <0.0001 | 1.18 | [0.95 ; 1.47] | 0.1349 |
| **Male donor** | 0.95 | [0.83 ; 1.10] | 0.5168 | - | - |  |
| **Positive donor CMV serology** | 1.29 | [1.11 ; 1.49] | 0.0007 | 1.11 | [0.93 ; 1.32] | 0.2500 |
| **Cold ischemia time** ≥**18 hours** | 1.37 | [1.18 ; 1.59] | <0.0001 | 0.95 | [0.79 ; 1.14] | 0.5834 |
| **Depleting induction at transplantation** | 1.02 | [0.86 ; 1.19] | 0.8473 | - | - |  |
| **Maintenance CNI treatment at transplantation** |  |  | 0.0535 | - | - |  |
| - **Ciclosporine (vs. Tacrolimus)** | 1.10 | [0.90 ; 1.34] |  | - | - |  |
| - **None (vs. Tacrolimus)** | 1.73 | [1.09 ; 2.76] |  | - | - |  |
| **CMV prophylaxis treatment at transplantation** | 1.09 | [0.91 ; 1.32] | 0.3545 | - | - |  |
| **Immunization pre-transplantation** |  |  | 0.2250 | - | - |  |
| - **anti-HLA no DSA (vs. Negative)** | 1.16 | [0.97 ; 1.38] |  | - | - |  |
| - **DSA (vs. Negative)** | 1.18 | [0.91 ; 1.54] |  | - | - |  |
| **HLA-A-B-DR mismatches > 4** | 0.84 | [0.69 ; 1.02] | 0.0841 | - | - |  |
| **Pregnancy history** | 0.77 | [0.62 ; 0.95] | 0.0148 | 0.75 | [0.49 ; 1.16] | 0.2008 |
| **Delayed graft function** | 1.85 | [1.58 ; 2.16] | <0.0001 | 1.20 | [1.00 ; 1.45] | 0.0524 |
| **At least one rejection** | 1.14 | [0.95 ; 1.36] | 0.1553 | - | - |  |
| **At least one CMV infection** | 1.47 | [1.20 ; 1.80] | 0.0002 | 1.18 | [0.93 ; 1.49] | 0.1765 |
| AGPN, acute graft pyelonephritis, CI, confidence interval; CMV, cytomegalovirus; DSA, donor-specific antibodies; HLA, human leucocyte antigens; HR, hazard ratio. | | | | | | |

**Table S4.** Results of the unadjusted and multivariable cause-specific time-dependent Cox models stratified on center studying the risk of occurrence of de novo DSA.

|  | **Unadjusted Cox models** | | | **Adjusted Cox model** | | |
| --- | --- | --- | --- | --- | --- | --- |
|  | **HR** | **95% CI** | **p-value** | **HR** | **95% CI** | **p-value** |
| **AGPN** |  |  | 0.5702 |  |  | 0.2474 |
| **One early (vs. None)** | 1.14 | [0.96 ; 1.36] |  | 1.02 | [0.82 ; 1.27] |  |
| **Two at least the first occurs early (vs. None)** | 1.15 | [0.84 ; 1.57] |  | 0.73 | [0.46 ; 1.16] |  |
| **Three or more at least the first occurs early (vs. None)** | 0.87 | [0.53 ; 1.44] |  | 0.92 | [0.49 ; 1.74] |  |
| **One late AGPN and no early (vs. None)** | 1.15 | [0.87 ; 1.52] |  | 1.27 | [0.89 ; 1.82] |  |
| **Two late AGPN and no early (vs. None)** | 0.82 | [0.50 ; 1.37] |  | 0.48 | [0.23 ; 0.99] |  |
| **Three or more late AGPN and no early (vs. None)** | 1.08 | [0.58 ; 2.02] |  | 0.81 | [0.32 ; 2.02] |  |
| **Recipient age** ≥**55 years** | 0.90 | [0.83 ; 0.98] | 0.0139 | 0.91 | [0.80 ; 1.03] | 0.1359 |
| **Retransplantation** | 1.42 | [1.27 ; 1.59] | <0.0001 | 1.19 | [1.03 ; 1.38] | 0.0197 |
| **Male sex** | 0.98 | [0.90 ; 1.06] | 0.6292 | - | - |  |
| **Kidney transplantation (vs SPK transplantation)** | 0.62 | [0.51 ; 0.76] | <0.0001 | 0.56 | [0.42 ; 0.76] | 0.0001 |
| **Renal replacement therapy** |  |  | 0.0012 |  |  | 0.0624 |
| - **Hemodialysis (vs. Preemptive transplantation)** | 1.11 | [0.99 ; 1.24] |  | 1.03 | [0.90 ; 1.19] |  |
| - **Peritoneal dialysis (vs. Preemptive transplantation)** | 0.84 | [0.70 ; 1.01] |  | 0.80 | [0.63 ; 1.01] |  |
| **History of diabetes** | 1.30 | [1.18 ; 1.43] | <0.0001 | 1.13 | [0.98 ; 1.30] | 0.1030 |
| **Recurrent initial nephropathy** | 0.94 | [0.85 ; 1.03] | 0.1633 | - | - |  |
| **Uro-nephrological history** | 1.08 | [0.95 ; 1.22] | 0.2626 | - | - |  |
| **Positive recipient CMV serology** | 1.14 | [1.04 ; 1.25] | 0.0051 | 0.99 | [0.88 ; 1.11] | 0.8127 |
| **Deceased donor** | 1.05 | [0.95 ; 1.17] | 0.3060 | - | - |  |
| **Donor age** ≥**55 years** | 0.90 | [0.83 ; 0.97] | 0.0087 | 0.98 | [0.86 ; 1.11] | 0.7338 |
| **Male donor** | 0.97 | [0.90 ; 1.05] | 0.4778 | - | - |  |
| **Positive donor CMV serology** | 1.02 | [0.94 ; 1.11] | 0.5674 | - | - |  |
| **Cold ischemia time** ≥**18 hours** | 0.99 | [0.90 ; 1.08] | 0.7553 | - | - |  |
| **Depleting induction at transplantation** | 1.95 | [1.76 ; 2.16] | <0.0001 | 1.35 | [1.19 ; 1.54] | <0.0001 |
| **Maintenance CNI treatment at transplantation** |  |  | <0.0001 |  |  | 0.2473 |
| - **Ciclosporine (vs. Tacrolimus)** | 0.69 | [0.61 ; 0.77] |  | 1.06 | [0.90 ; 1.24] |  |
| - **None (vs. Tacrolimus)** | 0.78 | [0.55 ; 1.09] |  | 0.75 | [0.51 ; 1.10] |  |
| **CMV prophylaxis treatment at transplantation** | 1.15 | [1.03 ; 1.28] | 0.0128 | 1.08 | [0.94 ; 1.23] | 0.2606 |
| **Immunization pre-transplantation** |  |  | <0.0001 |  |  | <0.0001 |
| - **anti-HLA no DSA (vs. Negative)** | 1.60 | [1.42 ; 1.80] |  | 1.57 | [1.36 ; 1.82] |  |
| - **DSA (vs. Negative)** | 6.86 | [5.93 ; 7.94] |  | 6.48 | [5.41 ; 7.76] |  |
| **HLA-A-B-DR mismatches > 4** | 1.44 | [1.30 ; 1.58] | <0.0001 | 1.37 | [1.20 ; 1.56] | <0.0001 |
| **Pregnancy history** | 1.13 | [1.01 ; 1.26] | 0.0271 | 1.01 | [0.89 ; 1.15] | 0.8572 |
| **Delayed graft function** | 1.14 | [1.03 ; 1.25] | 0.0110 | 0.97 | [0.85 ; 1.10] | 0.6063 |
| **At least one rejection** | 1.56 | [1.37 ; 1.77] | <0.0001 | 1.53 | [1.29 ; 1.81] | <0.0001 |
| **At least one rejection x At least one AGPN** |  |  |  | 1.10 | [0.74 ; 1.65] | 0.6349 |
| **At least one CMV infection** | 1.28 | [1.10 ; 1.49] | 0.0016 | 1.27 | [1.04 ; 1.54] | 0.0178 |
| AGPN, acute graft pyelonephritis, CI, confidence interval; CMV, cytomegalovirus; DSA, donor-specific antibodies; HLA, human leucocyte antigens; HR, hazard ratio. | | | | | | |

**Table S5.** Results of the multivariable linear mixed model of eGFR (ml/min/1.73m^2^) including random intercept and random slope when the baseline was set at 6 months post-transplantation.

|  | **Adjusted mean difference** | **95% CI** | **p-value** |
| --- | --- | --- | --- |
| **AGPN** |  |  | <0.0001 |
| **1 (vs. 0)** | -3.02 | [-4.53 ; -1.52] |  |
| **2 or more (vs. 0)** | -4.67 | [-7.51 ; -1.83] |  |
| **Recipient age** ≥**55 years** | -1.75 | [-2.83 ; -0.67] | 0.0015 |
| **Male sex** | 0.41 | [-1.27 ; 2.08] | 0.6349 |
| **Kidney transplantation (vs SPK transplantation)** | -10.27 | [-12.55 ; -7.99] | <0.0001 |
| **Renal replacement therapy** |  |  | 0.0028 |
| - **Peritoneal dialysis (vs. Hemodialysis)** | -0.59 | [-2.07 ; 0.89] |  |
| - **Preemptive transplantation (vs. Hemodialysis)** | -2.09 | [-3.28 ; -0.89] |  |
| **History of diabetes** | -0.07 | [-1.19 ; 1.06] | 0.9092 |
| **Deceased donor** | -4.22 | [-5.45 ; -3.00] | <0.0001 |
| **Donor age** ≥**55 years** | -11.95 | [-13.01 ; -10.88] | <0.0001 |
| **Male donor** | 4.55 | [3.68 ; 5.42] | <0.0001 |
| **Positive donor CMV serology** | 0.44 | [-0.46 ; 1.33] | 0.3402 |
| **Cold ischemia time** ≥**18 hours** | -0.40 | [-1.41 ; 0.60] | 0.4307 |
| **CMV prophylaxis treatment at transplantation** | 1.02 | [0.07 ; 1.97] | 0.0360 |
| **Immunization pre-transplantation** |  |  | 0.2063 |
| - **DSA (vs. anti-HLA no DSA)** | -0.86 | [-2.20 ; 0.49] |  |
| - **Negative (vs. anti-HLA no DSA)** | 0.38 | [-0.55 ; 1.31] |  |
| **HLA-A-B-DR mismatches > 4** | -0.24 | [-1.35 ; 0.86] | 0.6668 |
| **Pregnancy history** | -2.29 | [-4.11 ; -0.46] | 0.0140 |
| **Delayed graft function** | -5.51 | [-6.57 ; -4.44] | <0.0001 |
| **Rejection before 6 months post-transplantation** | -6.45 | [-7.62 ; -5.27] | <0.0001 |
| **CMV infection before 6 months post-transplantation** | -2.05 | [-3.47 ; -0.63] | 0.0048 |
| **Time from baseline to 1 year post-transplantation (months)** | 0.08 | [0.04 ; 0.13] | 0.0007 |
| **Time from 1 year post-transplantation (months)** | -0.10 | [-0.11 ; -0.09] | <0.0001 |
| **One AGPN x time from baseline (months)** | -0.01 | [-0.05 ; 0.03] | 0.4791 |
| **Two or more AGPN x time from baseline (months)** | -0.04 | [-0.12 ; 0.04] | 0.3491 |
| AGPN, acute graft pyelonephritis, CI, confidence interval; CMV, cytomegalovirus; DSA, donor-specific antibodies; HLA, human leucocyte antigens. | | | |

**Table S6.** Results of the multivariable linear mixed model of eGFR (ml/min/1.73m^2^) including random intercept and random slope when the baseline was set at 12 months post-transplantation.

|  | **Adjusted mean difference** | **95% CI** | **p-value** |
| --- | --- | --- | --- |
| **AGPN** |  |  | <0.0001 |
| **One early (vs. None)** | -3.19 | [-4.91 ; -1.47] |  |
| **Two or more at least the first occurs early (vs. None)** | -4.29 | [-6.93 ; -1.65] |  |
| **One late AGPN and no early (vs. None)** | -6.84 | [-9.89 ; -3.80] |  |
| **Two or more late AGPN and no early (vs. None)** | -12.50 | [-18.74 ; -6.27] |  |
| **Recipient age** ≥**55 years** | -1.35 | [-2.51 ; -0.20] | 0.0217 |
| **Male sex** | 0.43 | [-1.38 ; 2.23] | 0.0499 |
| **Kidney transplantation (vs SPK transplantation)** | -9.02 | [-11.46 ; -6.58] | <0.0001 |
| **Renal replacement therapy** |  |  | NA |
| - **Peritoneal dialysis (vs. Hemodialysis)** | -0.14 | [-1.72 ; 1.44] |  |
| - **Preemptive transplantation (vs. Hemodialysis)** | -1.60 | [-2.87 ; -0.32] |  |
| **History of diabetes** | -0.22 | [-1.43 ; 0.99] | 0.7164 |
| **Deceased donor** | -4.00 | [-5.31 ; -2.68] | <0.0001 |
| **Donor age** ≥**55 years** | -12.01 | [-13.15 ; -10.87] | <0.0001 |
| **Male donor** | 4.35 | [3.42 ; 5.28] | <0.0001 |
| **Positive donor CMV serology** | 0.65 | [-0.32 ; 1.62] | 0.1900 |
| **Cold ischemia time** ≥**18 hours** | -0.25 | [-1.33 ; 0.83] | 0.6467 |
| **CMV prophylaxis treatment at transplantation** | 1.02 | [-0.00 ; 2.03] | 0.0410 |
| **Immunization pre-transplantation** |  |  | NA |
| - **DSA (vs. anti-HLA no DSA)** | -0.81 | [-2.26 ; 0.63] |  |
| - **Negative (vs. anti-HLA no DSA)** | 0.89 | [-0.11 ; 1.88] |  |
| **HLA-A-B-DR mismatches > 4** | -0.25 | [-1.43 ; 0.93] | 0.6789 |
| **Pregnancy history** | -2.60 | [-4.56 ; -0.64] | 0.0093 |
| **Delayed graft function** | -5.22 | [-6.37 ; -4.07] | <0.0001 |
| **Rejection before 12 months post-transplantation** | -6.81 | [-7.97 ; -5.65] | <0.0001 |
| **CMV infection before 12 months post-transplantation** | -2.56 | [-3.89 ; -1.23] | 0.0002 |
| **Time from baseline** | -0.10 | [-0.11 ; -0.09] | <0.0001 |
| **One early AGPN x time from baseline** | 0.01 | [-0.03 ; -0.03] | 0.0079 |
| **Two or more at least the first occurs early x time from baseline** | -0.07 | [-0.14 ; 0.00] | 0.0534 |
| **One late AGPN and no early x time from baseline** | 0.05 | [-0.03 ; 0.12] | 0.2162 |
| **Two or more late AGPN and no early x time from baseline** | -0.05 | [-0.19 ; 0.09] | 0.4901 |
| AGPN, acute graft pyelonephritis, CI, confidence interval; CMV, cytomegalovirus; DSA, donor-specific antibodies; HLA, human leucocyte antigens. | | | |

**Table S7.** Results of the multivariable linear mixed model of eGFR (ml/min/1.73m^2^) including random intercept and random slope when the baseline was set at 24 months post-transplantation.

|  | **Adjusted mean difference** | **95% CI** | **p-value** |
| --- | --- | --- | --- |
| **AGPN** |  |  | <0.0001 |
| **One early (vs. None)** | -1.94 | [-4.06 ; 0.18] |  |
| **Two or more at least the first occurs early (vs. None)** | -4.53 | [-7.48 ; -1.57] |  |
| **One late AGPN and no early (vs. None)** | -4.89 | [-7.49 ; -2.29] |  |
| **Two or more late AGPN and no early (vs. None)** | -8.56 | [-12.69 ; -4.43] |  |
| **Recipient age** ≥**55 years** | -0.58 | [-1.93 ; 0.76] | 0.3961 |
| **Male sex** | 1.63 | [-0.46 ; 3.73] | 0.3842 |
| **Kidney transplantation (vs SPK transplantation)** | -9.38 | [-12.34 ; -6.43] | <0.0001 |
| **Renal replacement therapy** |  |  | NA |
| - **Peritoneal dialysis (vs. Hemodialysis)** | 0.25 | [-1.57 ; 2.06] |  |
| - **Preemptive transplantation (vs. Hemodialysis)** | -0.92 | [-2.39 ; 0.54] |  |
| **History of diabetes** | -1.25 | [-2.68 ; 0.18] | 0.0869 |
| **Deceased donor** | -3.96 | [-5.47 ; -2.44] | <0.0001 |
| **Donor age** ≥**55 years** | -12.57 | [-13.89 ; -11.25] | <0.0001 |
| **Male donor** | 3.85 | [2.77 ; 4.93] | <0.0001 |
| **Positive donor CMV serology** | 0.62 | [-0.51 ; 1.75] | 0.2837 |
| **Cold ischemia time** ≥**18 hours** | -0.11 | [-1.35 ; 1.13] | 0.8627 |
| **CMV prophylaxis treatment at transplantation** | 0.63 | [-0.55 ; 1.81] | 0.3525 |
| **Immunization pre-transplantation** |  |  | NA |
| - **DSA (vs. anti-HLA no DSA)** | -1.03 | [-2.73 ; 0.66] |  |
| - **Negative (vs. Anti-HLA no DSA)** | 0.26 | [-0.89 ; 1.42] |  |
| **HLA-A-B-DR mismatches > 4** | -0.03 | [-1.41 ; 1.34] | 0.9647 |
| **Pregnancy history** | -1.56 | [-3.83 ; 0.72] | 0.1798 |
| **Delayed graft function** | -4.45 | [-5.79 ; -3.11] | <0.0001 |
| **Rejection before 24 months post-transplantation** | -7.41 | [-8.67 ; -6.14] | <0.0001 |
| **CMV infection before 24 months post-transplantation** | -3.00 | [-4.50 ; -1.50] | 0.0001 |
| **Time from baseline** | -0.10 | [-0.11 ; -0.09] | <0.0001 |
| **One early AGPN x time from baseline** | -0.01 | [-0.06 ; 0.04] | 0.6874 |
| **Two or more at least the first occurs early x time from baseline** | -0.06 | [-0.13 ; 0.02] | 0.1261 |
| **One late AGPN and no early x time from baseline** | -0.02 | [-0.08 ; 0.04] | 0.5755 |
| **Two or more late AGPN and no early x time from baseline** | -0.01 | [-0.10 ; 0.08] | 0.8580 |
| AGPN, acute graft pyelonephritis, CI, confidence interval; CMV, cytomegalovirus; DSA, donor-specific antibodies; HLA, human leucocyte antigens. | | | |
